# Neurophysiological correlates of automatic integration of voice and gender information during grammatical processing

**DOI:** 10.1038/s41598-022-14478-2

**Published:** 2022-07-30

**Authors:** Maria Alekseeva, Andriy Myachykov, Beatriz Bermudez-Margaretto, Yury Shtyrov

**Affiliations:** 1 Centre for Cognition and Decision Making, Institute for Cognitive Neuroscience, Higher School of Economics, Moscow, Russian Federation; 2 Department of Psychology, Northumbria University, Newcastle-upon-Tyne, UK; 3 Departamento de Psicología Básica, Psicobiología y Metodología de las Ciencias del Comportamiento, Instituto de Integración en la Comunidad - INICO, Facultad de Psicología, Universidad de Salamanca, Salamanca, Spain; 4 Center of Functionally Integrative Neuroscience (CFIN), Department of Clinical Medicine, Aarhus University, Aarhus, Denmark

**Keywords:** Language, Perception

## Abstract

During verbal communication, interlocutors rely on both linguistic (e.g., words, syntax) and extralinguistic (e.g., voice quality) information. The neural mechanisms of extralinguistic information processing are particularly poorly understood. To address this, we used EEG and recorded event-related brain potentials while participants listened to Russian pronoun–verb phrases presented in either male or female voice. Crucially, we manipulated congruency between the grammatical gender signaled by the verbs’ ending and the speakers’ apparent gender. To focus on putative automatic integration of extralinguistic information into syntactic processing and avoid confounds arising from secondary top-down processes, we used passive non-attend auditory presentation with visual distraction and no stimulus-related task. Most expressed neural responses were found at both early (150 ms, ELAN-like) and late (400 ms, N400-like) phrase processing stages. Crucially, both of these brain responses exhibited sensitivity to extralinguistic information and were significantly enhanced for phrases whose voice and grammatical gender were incongruent, similar to what is known for ERPs effects related to overt grammatical violations. Our data suggest a high degree of automaticity in processing extralinguistic information during spoken language comprehension which indicates existence of a rapid automatic syntactic integration mechanism sensitive to both linguistic and extralinguistic information.

## Introduction

During linguistic communication, we do not only rely on linguistic information as such (i.e., phonology, lexical semantics, grammar, etc.) but also make use of extralinguistic information provided by the speaker and by the conversation context (see for discussion^1,2^; for review^3^). Some of this information can be extracted from the speaker’s voice, and it may be particularly relevant for interpreting the message as it projects speaker-related characteristics, such as their identity, age, gender, and emotional state. Existing evidence suggests that this information can directly modulate sentence processing. For example, listener’s expectations about the speaker’s language can help decode the speech signal and predict upcoming information (see for general discussion^4^ and for example of predictive processing^5^). Yet, although many studies examined different psycho- and neurolinguistic processes underpinning language comprehension, little is known about the involvement of speaker-related features during this process.

The speaker’s voice provides the listener with a quick and reliable source of information that can facilitate sentence comprehension^6^. Among key characteristics which can be identified from the speaker’s voice is their apparent gender^7–12^. Voice gender is known to be processed by distributed brain networks; for instance, Junger et al.^9^ registered voice gender processing correlates in cingulate cortex and bilateral inferior frontal gyri, with activation particularly increased for opposite sex in fronto-temporal regions relatively early. This result suggests that, when comprehending the message, the listener takes into consideration both their own and the speaker’s gender at the first stages of voice processing. Furthermore, many languages mark the grammatical gender explicitly, adding another source of gender information and further complicating the interplay between these factors. Attempts to investigate how the listener integrates gender information from these different sources have shown, for example, that listeners process sentences faster when the grammatical gender is congruent with their own gender^13^, resulting in shorter reaction times in congruent conditions. An ERP study of semantically gendered words also showed an early response—an enhanced mismatch negativity (MMN)—to words whose semantic gender matched the gender of the speaker^14^, presumably reflecting facilitated activation of the underlying memory traces. Furthermore, an interaction between the speaker’s and the listener’s genders during sentence comprehension was documented as an early (at around 150–250 ms) increase of the MMN amplitude in listeners presented with opposite gender voices, compared to gender-matching stimuli^14^.

Other ERP studies have reported diverse neurophysiological responses to inconsistencies between the message meaning and the speaker’s representation, typically manifest as a modulation of the N400 and/or P600 components^15–17^. Different patterns of ERP results reported in these studies are likely related to the nature of the mismatch manipulations used. For instance, whereas the P600 component is typically associated with a reanalysis/repair of syntactic incongruences and grammatical violations^18^, in experiments modulating the speaker’s voice it can also be elicited by the violations of the stereotypical noun roles in the absence of grammatical incongruencies as such (e.g., “*face powder*” or “*fight club*”, produced by male and female voices, respectively^16^) as well as the general assumptions based on the pronoun processing during sentence comprehension^19^. In contrast, the semantically-related N400 effect has been typically found for the semantic-pragmatic incongruences (e.g., “*I am going to the night club*” by child’s voice^17^).

Interestingly, these ERP effects offer support to two models of pragmatic language comprehension—the standard, two-step model and the one-step model. The two-step model claims that listeners compute meaning first, in isolation, and that the communicative context is considered at the second stage (speaker’s information, in particular^16,20^), as reflected in the late P600 responses. More recent findings showed, however, that this pragmatic (extralinguistic) integration is likely happening in a single-step manner already during semantic processing, as reflected in the N400 effect^17,21^. Nevertheless, other studies also reported the overlap of both processing stages, showing an N400 effect elicited by expectation error and a late P600 effect for overall reanalysis of this expectation^22^.

Understanding how gender information is integrated by the listeners is particularly important when one considers the differences in how different languages signal grammatical gender. In some languages, such as in English, Finnish or Mandarin, overt grammatical gender marking is almost completely absent. Many other languages, such as Slavic languages, explicitly mark grammatical gender in nouns, verbs, and adjectives, often in a complicated interdependent manner. Russian is one of such languages, offering an optimal testbed for investigating linguistic and extralinguistic gender integration. As far as we know, there is only one study addressing this question in a Slavic language: using Slovak, Hanulíková Carreiras^23^ found that, during an active-listening task, the integration of speaker-related information and morphosyntactic information occurred rather late during complex sentence processing. Additionally, a conflict between the speaker’s and the word’s genders (e.g., “*I-* $stole_{MASC}$* plums*” in female voice) was reflected in the modulation of the N400 component. Given that N400/LAN modulations have been consistently found for morphosyntactic violations, in particular for number, person, and gender agreement, as well as in phrase structure violations (e.g.,^24^, see also for review^25^), this result may suggest that extralinguistic information is directly integrated during online (morpho)syntactic processing (such as speaker’s sex converted into subject’s gender in (morpho)syntactic processing). However, N400 is also known to be related to conscious top-down controlled integration of linguistic information^24,26^. Indeed, in the study described above, the participant’s overt attention to the stimuli was required, and the effect generally appeared rather late in the comprehension processes. Thus, the question of whether such findings reflect the involvement of genuine online parsing mechanisms or secondary post-comprehension processes (such as repair and reanalysis^24,27^) still remains unsolved. Importantly, syntactic parsing has been shown to commence much earlier and to take place in a largely automatic fashion, as demonstrated in studies focused on early left-anterior negativity (ELAN) or syntactic MMN. In particular, ELAN modulation around 200 ms or earlier has been reported during outright violations of the obligatory structure, reflecting an automatic early analysis of the syntactic structure like phrase structure errors^28–31^, and it is considered to reflect the brain’s response to the word category violations.

As the onset of ELAN may be quite early (100 ms), some researchers have questioned whether this component actually reflects syntactic processing, since even lexical access has been argued to commence around 200 ms post-stimulus onset^32,33^. Some authors have also argued that ERPs occurring before the onset of a critical word may introduce spurious shifts in downstream ERPs when applying a baseline correction, leading to artifactual ELAN-like activity^34^. On the other hand, it has also been shown that language comprehension system can predict the syntactic structure from some characteristics at an initial stage thus leading to very early syntactically-related activations^35^. These processing events are often referred to as basic syntactic parsing, starting with pre-processing of the syntactic structure, and it is often assumed that they necessarily precede semantic processing^36,37^. Finally, early first-pass syntactic parsing stage is believed to have a high degree of automaticity as it has been shown to be independent of focused attention on the input, taking place even when the subject is not focused on the language stimuli^29^.

Similar early morphosyntactic effects have been found reflected in the MMN response. This component, originally related to acoustic change detection and auditory short-term memory^38^ is also considered to index higher-level language processes^39,40^. For instance, the so-called syntactic MMN (sMMN) was found to be elicited by syntactic violations in the left-hemispheric language systems^30,41–44^. Previous studies found that whenever the deviant sequence included a verb person/suffix agreement violation (e.g., “we *walks”), it caused a larger sMMN in comparison with the same verb presented in agreement with the pronoun (“*he walks*”). These studies suggest that the auditory sMMN reflects similar early automatic processes as ELAN, based on the pre-activation of morphosyntactically plausible representations^45^. Crucially, all of these effects were registered when participants’ attention was diverted away from the auditory stimuli, typically using an active visual task. Furthermore, when directly comparing conditions with attention focused on vs. diverted away from the syntactic inputs, it was found that this early sMMN/ELAN deflection was not affected by attention allocation until approximately 200 ms, implying a strong degree of automaticity in early syntactic parsing^44^. Such early automatic (morpho)syntactic activity may reflect processes associated with the listener’s attempts to maintain efficient alignment with the speaker and associated prediction (priming) mechanisms^46^. As such, they may have to rely upon activating syntactic templates and junctions as early as possible and often in a highly predictive and automatized fashion^42,47^.

Although such early automatic (morpho)syntactic processing has been repeatedly demonstrated in neurophysiological research, it remains unclear whether the listener’s brain makes use of extralinguistic speaker-related information during syntactic parsing in a similarly rapid automated fashion. It has been suggested that the integration of gender information during speech might happen at the sub-lexical and lexical levels^48^. Given the reflection of these processes in the MMN responses^14^, it stands to reason that such gender integration takes place at the early stages of morphosyntactic processing, although the experimental evidence for this is still scarce.

Furthermore, studies focused on extralinguistic processing are also limited with regard to the language in which the materials are presented. Languages with a shallow inflectional system (such as English, the most often used language in psycho- and neurolinguistic literature) lack key grammatical features to tackle the processing of speaker-word gender incongruences. The present study is aimed at filling these gaps by examining the neurophysiological correlates of automatic extralinguistic processing using subject-verb agreement in a language with overt gender marking (Russian) under non-attend design while carefully balancing acoustic, psycholinguistic and voice-related properties of the stimuli.

In particular, we investigated how and when linguistic and extralinguistic gender information (subject’s gender), such as speaker-dependent voice congruency, is integrated during sentence processing. For this purpose, we developed an auditory passive presentation protocol, similar to those previously employed in both sMMN and ELAN studies (see e.g.,^30^). Participants were presented with short auditory phrases while their attention was diverted away from the auditory stimuli to a primary visual task. Furthermore, by using male and female voices that either matched or mismatched with the grammatical gender agreement of the linguistic stimuli (e.g., “*I *$walked_{MASC}$” recorded in male or female voice), we directly assessed the integration of voice-related extralinguistic information (subject’s gender as perceived through voice) into automatic morphosyntactic processes. Since both grammatical verb-gender agreement parsing and gender-voice processing may occur quite early^14^, one may hypothesize an interaction and integration between the two processes, ensuring the efficiency of early automatic morphosyntactic analysis.

Our hypotheses were motivated by previous findings showing early automatic morphosyntactic and speaker’s gender processing starting before 200 ms [morphosyntactic^41–43,49^; speaker’s gender^14^]. In case of simultaneous integration of extralinguistic information expressed by speaker’s gender (in)congruency with standard automatic morphosyntactic processing based on pre-activation of a particular morphosyntactic feature by preceding speaker’s gender, we expected to observe a similarly early automatic ELAN-like modulation in the voice-gender mismatch conditions, supporting concurrent one-step processing of different information types. If the extralinguistic information integration, in line with two-step pragmatic models, is slower than early automatic morphosyntactic processing, no effects should be expected at early stages, either only modulating later responses in the N400-P600 range (similar to^23^) or showing no modulation at all in our non-attend design.

## Methods

### Participants

We recruited 37 right-handed participants (17 males, age range 19-32 years, mean age = 22.55, SD = 3.1) to take part in the experiment. All of them were Russian native speakers with no history of neurological or psychiatric disorders, normal or corrected-to-normal vision and normal hearing. All participants gave their informed written consent prior to taking part in the experiment and received monetary remuneration for their time in accordance with the university compensation rules. The research was approved by the HSE University Psychology Department Ethics Committee, Moscow, Russia.

### Materials

**Figure 1:**
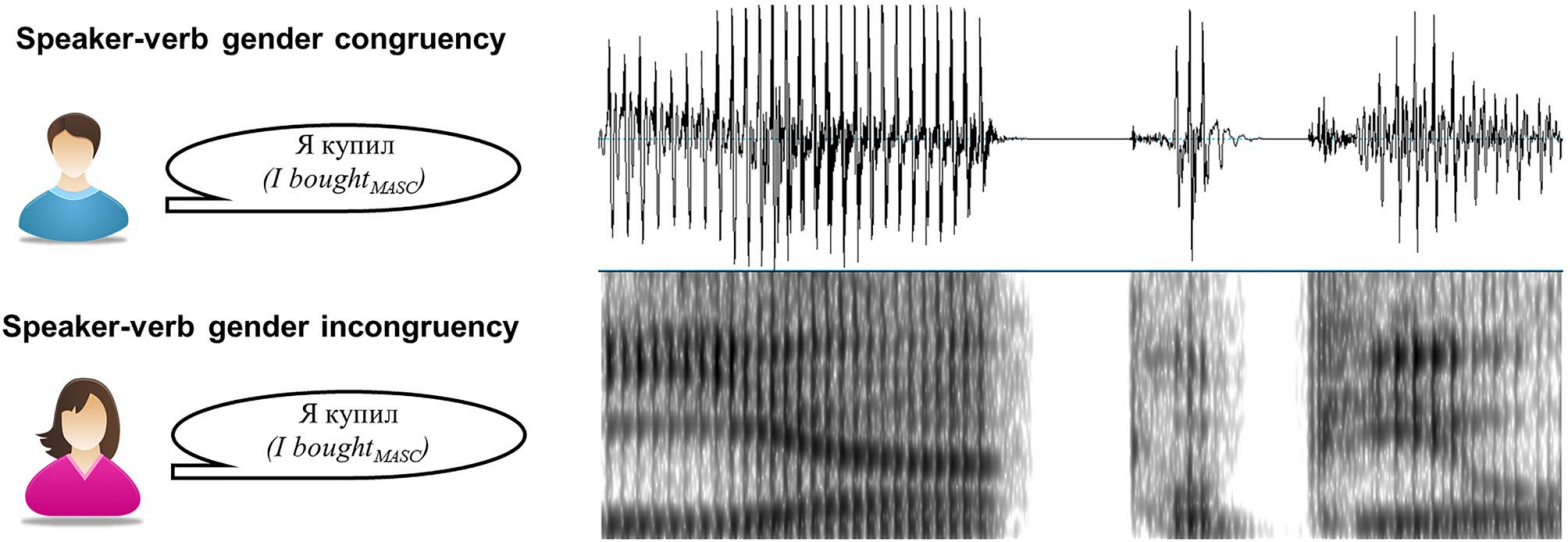
Example of an experimental stimulus (“*ja kupil; I* $bought_{MASC}$”) presented in both congruent and incongruent conditions, together with its waveform and spectrogram.

Ten Russian verbs comprised of 5 phonemes - *kupil* ([kʊ$p^j$ˈ il], *bought*), *velel* ([$v^j$iˈ$l^j$el], *ordered*), *zapel* ([zɐˈ$p^j$el], *sang*), *nadel* ([nɐˈ$d^j$el], *put on*), *pobil* ([pɐˈ$b^j$il], *broke/beat*), *zasel* ([zɐˈ$s^j$el], *sat*), *popal* ([pɐˈ pal], *got*), *polil* ([pɐˈ$l^j$il], *watered*), *pozhal* ([pɐˈʐal], *shook*), *sumel* ([sʊˈ$m^j$el], *could*), all combined with first-person singular pronoun *ja* (*I*), were selected as experimental materials. All verbs were used in past tense (as past tense in Russian is gender-marked, whereas present and future tenses are not), thus consisting of four-phonemes and masculine past tense suffix -*l* ([-l]). In order to allow speaker’s gender manipulation, all sentences were presented in two versions—in both female and male voice, which only differed in the voice-gender agreement, but not in grammatical agreement. Therefore, all phrases were grammatically correct in terms of their subject-verb agreement (see stimuli presentation on Fig. 1). By combining the pronoun with verb phrases (e.g., “*ja kupil*” [ja kupil], “$I bought_{MASC}$”) and voice, we constructed 2 sets of 10 sentences. Thus, the verb’s morphology (suffix/ending) provided information not only about the syntactic congruency of the phrase but also about the match between the speaker’s perceived voice gender and the grammatical—masculine—gender of the verb. Hence identical verbs appeared in congruent and incongruent voice-gender conditions, differing only in the presentation voice. Sentences were synthesized with the help of Voice Reader Home 15 (Linguatec) selecting male and female Russian speakers.

All experimental stimuli had high form and bigram frequencies (extracted from The Russian National Corpus, https://ruscorpora.ru/; see Appendix A). To minimize acoustic variability between stimuli, which could affect brain responses, pronouns and verbs were generated separately and then cross-spliced into sentences by means of Audacity v2.3.0 software (Audacity Team). The pronoun length as well as pauses between the phrase constituents and the final verbal suffix were equal across all stimuli. Unnatural transitions and/or artificial sounds in the stimuli were prevented. By manipulating intensity, amplitude, and duration we created stimuli with virtually identical acoustic properties. Most importantly, the critical segment of interest, namely the past tense suffix, was matched in length across all experimental stimuli. All the auditory stimuli were set in mono using .wav format with file duration of approximately 1000 ms (see Appendix B for details).

### Design and procedure

Participants were presented with the stimuli at a comfortable hearing level binaurally via insert earphones. They were seated in an electrically and acoustically insulated chamber and instructed to watch a silent film throughout the duration of the experiment (Wallace and Gromit: The Curse of the Were-Rabbit), and to pay no attention to the auditory stimuli. The film was presented on a 28-inch Samsung-U28D590D LCD monitor with 1920 x 1080 resolution at 100 Hz refresh rate. Auditory stimuli were programmed and presented using E-prime v2.0 software (Psychology Software Tools, USA). During the entire experiment, the brain’s electrical activity was recorded using EEG (see details below).

We used 10 sentences presented in two voices during the experiment, each repeated 20 times in pseudorandom fashion, therefore 2 × 10 × 20 = 400 total trials. The average stimulus onset asynchrony (SOA) was 1050 ms, jittered to range from 1000 to 1100 ms. The sequence was subdivided into 4 blocks, 3.5 min each, to reduce participants’ fatigue. An initial training block with 10 trials was carried out to familiarize participants with the paradigm and consisted of similar verbs (but not identical to the experimental materials). A pseudo randomization of stimuli was established in each block such that the same phrase could not occur twice in a row.

There were self-regulated breaks between blocks, when participants were asked to fill a multiple (5) choice questionnaire about the content of the film. This ensured that participants indeed paid attention to the film and not to the auditory stimuli. The questionnaire contained a total of 20 questions with 5 questions per block. At the end of the task, participants were asked to carry out a word recognition task in order to provide a behavioral verification of the passive presentation of audio stimuli and to determinate that they were sufficiently distracted from the auditory input. It consisted of 10 experimental verb forms plus 20 foils (experimental verbs in another form and filler verbs). Participants had to read and indicate whether the item appeared in the experiment. The total duration of the experiment was 50 min excluding EEG set-up and preparation.

### EEG recording and pre-processing

The brain’s activity was continuously recorded by means of 128 active electrodes and amplified using an actiCHamp EEG system (Brain Products GmbH, Germany). Electrodes were mounted in an elastic cap (EasyCap, Brain Products GmbH, Germany) following the standard 10%-20% EEG configuration system. During the recording, EEG signals were sampled at 1000 Hz and a notch filter was applied at 50 Hz to remove line noise. Cz (vertex) was used as a reference electrode. Three electrodes were used for electrooculogram (EOG) recordings—two of them were placed on left and right canthi of the participants’ eyes for recording of horizontal ocular activity and another one under the participant’s right eye, for the recording of vertical ocular activity. Impedances were always kept below 10 k$\Omega$. Stimulus markers were sent to the recording computer at the onset of the past tense verb suffix using E-prime software.

Preprocessing of the EEG data was conducted using BrainVision Analyzer 2.1.2 software (Brain Products GmbH, Germany). First, high- and low-pass filters were applied at 0.1 and 30 Hz, respectively, following filtering parameters applied in similar studies^50,51^. New bipolar horizontal and vertical EOG signals were computed by subtracting differences between monopolar channels (Right minus Left for HEOG and lower VEOG minus FP2, respectively). Before artifact rejection, raw data were inspected with $\pm 100\,\upmu$V amplitude and 100 $\upmu$V of max-min difference as the artifact criteria within individual channels. This step ensured the detection of channels with sustained bad signal throughout recordings and their correction at a later step. Following raw data inspection, ocular correction ICA was used in order to remove all independent components reflecting ocular activity (saccades and blinks); ocular channels and bad channels were not included into the ICA procedure. Then, triangular topographic interpolation was carried out to recover bad channels detected previously during raw data inspection. EEG data were epoched per participant and per condition (congruent and incongruent) using the 100 ms before and 800 ms after the onset of the past tense verb suffix. The onset time of the verbal suffix was individually marked in the data, using triggers with time-locking specific to suffix onset in each phrase, to avoid any smearing of responses and to focus on the suffix identification points when the critical processing of gender information could commence. The 100 ms pre-suffix interval was used for baseline correction. Following this, artifact rejection was applied to all epochs, with the same criteria as for the raw data inspection. Then, an average reference was applied, with the EEG signal in each epoch re-referenced to the mean activity in all EEG channels. Finally, the remaining artifact-free epochs were averaged per each participant and condition in order to compute ERPs. The number of epochs used per each condition in each subject was at least 90% of trials, i.e., 180 out of 200 (congruent condition: mean = 195.14, range = 180-200, SD = 7.08, incongruent condition: mean =195.88, range = 180-200, SD = 6.82; differences not significant; data from 3 participants were deleted due to lower number of epochs following this criteria).

## Data analysis

**Figure 2:**
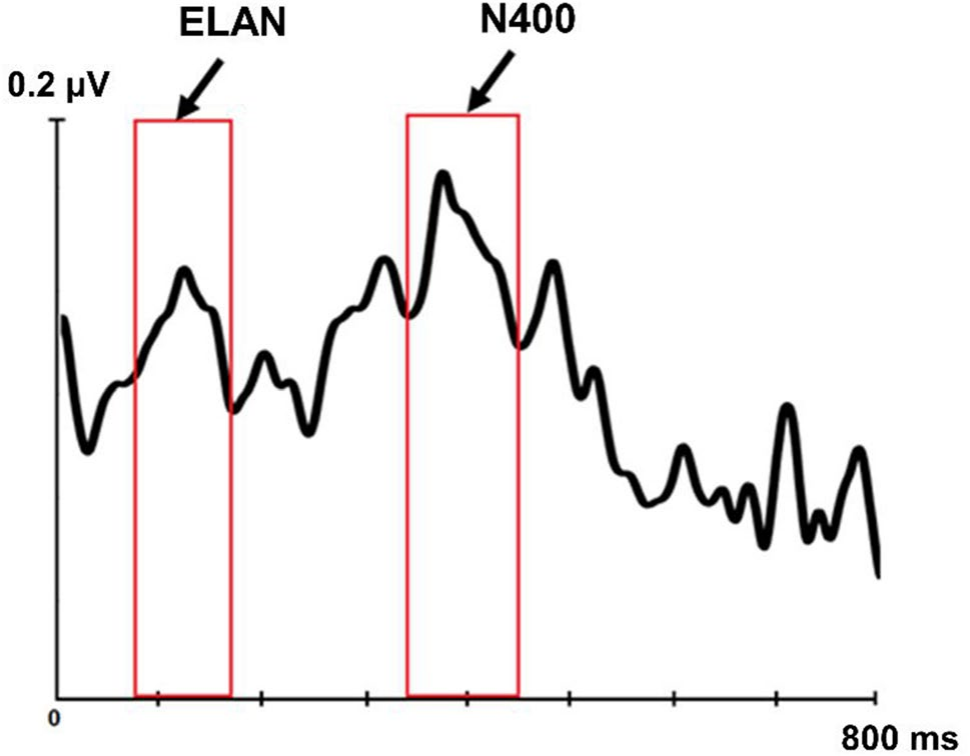
Global Field Power (GFP) waveform, computed across all participants, electrodes, stimuli, and conditions. Red bars highlight two main peaks in the overall neural activity along the ERP segment, maximal at around 150 ms and 400 ms post-suffix onset.

For unbiased data-driven analysis, overall activation was first quantified as the global field power (GFP) of the ERP responses across all participants, scalp electrodes, stimuli, and conditions. To this end, the grand average response was first calculated across all conditions and stimuli collapsed and then the root-mean-square was calculated on the sum of squared amplitudes across all electrodes. Finally, the most prominent peaks in the global responses were identified. This is believed to be optimal for approaching data in an unbiased way^52^ by focusing on the periods of largest neuronal activity overall and thus avoiding double-dipping in dataset comparisons. These GFP curves manifested 2 distinct peaks of different length^24^, maximal at 150 ms and 400 ms after the onset of the past tense verb suffix (see Fig. 2). Thus, based on the GFP waveform (as well as the previous literature which typically uses shorter and longer analysis windows for early and late peaks, respectively), we selected two time windows centered on these main peaks (130–170 ms and 350–450 ms) for further analysis; we extracted window-mean amplitudes from each participant, electrode, and condition and subjected them to further statistical analyses.

Repeated-measures analyses of variance (rmANOVA) were carried out in order to analyze differences in mean activity between congruent and incongruent conditions, separately for each time window. To this end, window-mean amplitude data from an array of 64 electrodes were included into the statistical analysis, broadly distributed over frontal, central, and posterior areas where auditory ERPs are typically most pronounced. The selection of these regions for the analysis followed previous findings related to auditory syntactic processing. These electrodes were divided into eight topographical lines (F, FFC, FC, FCC, C, CCP, CP and CPP) with eight electrodes in each line. The mean activity for each electrode in each line was submitted to a $2 \times 8 \times 8$ rmANOVA with factors Voice congruency (congruent vs. incongruent) $\times$ Anterior-Posterior (8 horizontal lines) $\times$ Laterality (8 electrodes—left to right). All *p* values were corrected for non-sphericity using Greenhouse–Geisser correction where appropriate. Effects reaching significance were followed-up with post-hoc-tests, employing Bonferroni correction for multiple comparisons.

## Results

### Behavioral data

Behavioral data analyses included mean scores from each participant for the film questionnaire and the verb recognition task in order to assess their compliance with the task. Ratings obtained for the film questionnaires indicated that participants were paying attention to the video during auditory presentation (mean correct answers = 19.31 out of 20, SD = 0.3; one-sample *t* test: *t* = 4.31, *p* = 0.0001). Conversely, ratings for the verb recognition task showed poor memory of the auditory stimuli (experimental verb form: mean= 4.47 out of 10, SD = 1.4; *t* = 2.08, *p* = 0.045; experimental verb in another form: mean = 1.56 out of 5, SD = 1.2; *t* = 0.37, *p* = 0.71; filler verb: mean = 1.66 out of 15, SD = 1.5; *t* = 2.01, *p* = 0.055). Since all participants were compliant with the task, paying attention to the film and not to the auditory stimulation data, all participants’ ERP data were used in further analysis.

### ERP data

#### ELAN-like component (150 ms)

**Figure 3:**
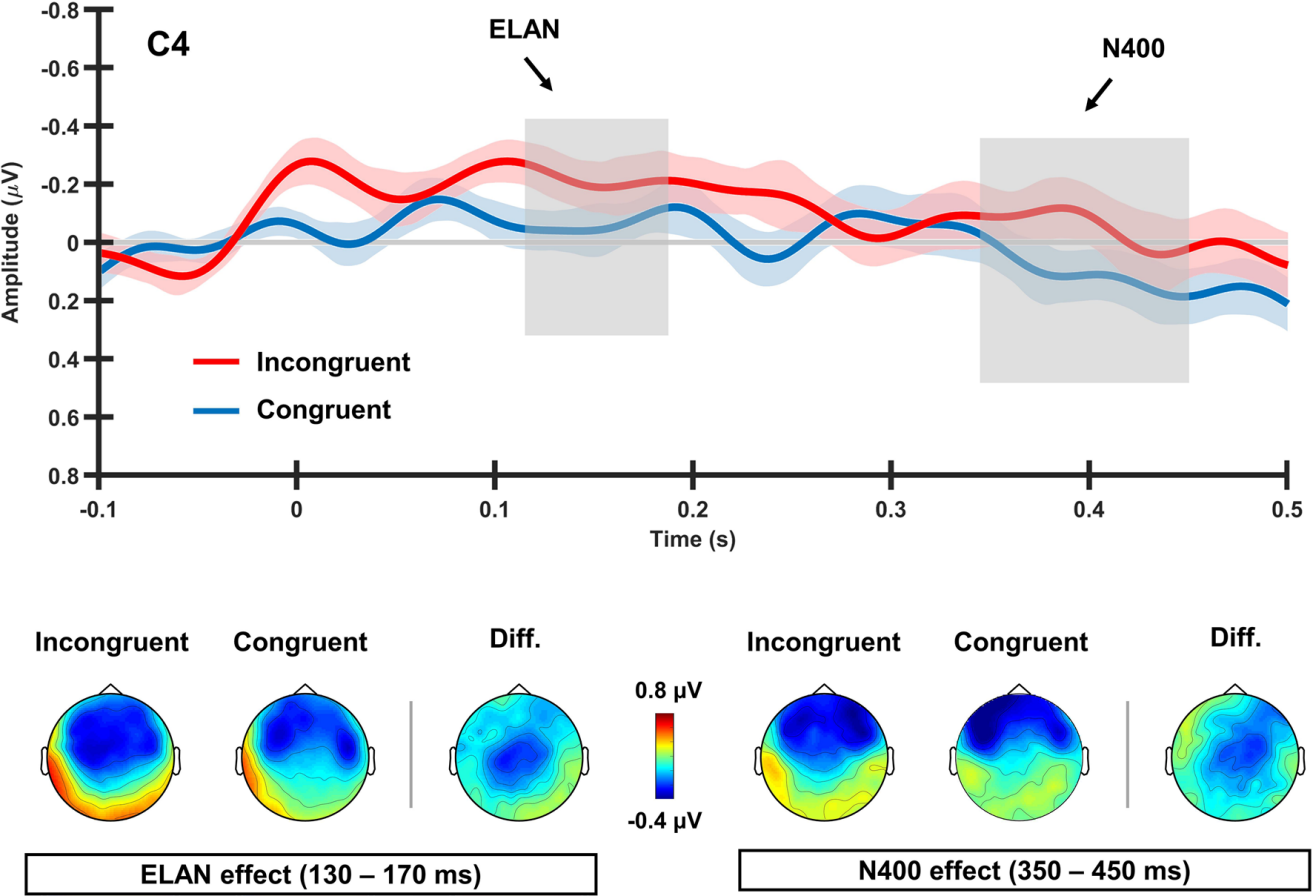
Averaged ERP waveforms and topographic maps for Congruent and Incongruent conditions. Arrows indicate early and late Voice Congruency effects at 150 ms and 400 ms after verb-suffix onset on a representative scalp site (C4), compatible with ELAN-like and N400-like components, respectively.

Figure 3 presents ERP waveforms and topographic distribution of brain responses at the early time window of 130–170 ms where an ELAN-like response was registered. rmANOVA indicated a significant three-way interaction between Voice Congruency and topographical Anterior-Posterior and Laterality factors ($\textit{F}_{49,1764}$= 2.634, *p* = 0.006, ${\eta }^{2}$ = 0.068). Post-hoc comparisons indicated more negative responses for incongruent stimuli at fronto-central, central, and centro-posterior regions (FFC1h, FFC2h, FC1, FC2, FCC1h, FCC2h, C1, C2, C4, CCP3h, CCP1h, CCP2h, CCP4h, CP1, CP2; see Appendix C for follow-up statistic results across topographic regions; see Appendix D for additional ERP waveforms showing the ELAN-like effect across different topographies).

#### N400 component (400 ms)

Analysis of the Voice Congruency effect at the later time window (350–450 ms) revealed a significant three-way interaction between Voice Congruency, Anterior-Posterior and Laterality ($\textit{F}_{49,1764}$= 2.072, *p* = 0.024, ${\eta }^{2}$ = 0.054). Post-hoc comparisons showed stronger negative responses for the stimuli incongruent with the speaker’s gender at the right fronto-central and central regions (FC4, FCC4h, FCC6h, C4; see Fig. 3; see Appendix E for follow-up statistic results across topographic regions; see Appendix F for additional ERP waveforms showing the N400-like effect across different topographies).

## Discussion

The current EEG study used Russian—a morphologically explicit language with a rich gender-marking system—to investigate online neural dynamics elicited by automatic syntactic processing under the influence of extralinguistic gender information provided by the speaker’s voice. For that purpose, a passive auditory presentation protocol was used, presenting a set of controlled first-person pronoun–verb phrases, where the verb gender marking was either congruent or incongruent with the responses to spoken stimuli. Analysis of the speaker-voice congruency contrast revealed both early and late ERP effects indicating increased negativity in the voice-gender incongruent conditions. In what follows we briefly summarize and discuss the main findings of the study.

A significant incongruency effect was found as early as 130–170 ms after recognition of grammatical gender (suffix onset), likely driven by the identification of verb’s morphology. This latency broadly corresponds to the time window of the well-known ELAN component, which typically shows an enhancement of the negativity for syntactically incongruent vs. congruent stimuli over left anterior channels^24,31^. Furthermore, ad hoc analyses of the time window preceding the ELAN effect suggest that it starts somewhat earlier (with a significant divergence already at 110–130 ms, *p *< 0.05), which is in agreement with the other studies showing the relatively early onset of morphosyntactic processing (e.g., sMMN^41^). It may therefore be that the present early ERP modulation reflects concurrent processing and integration of morphosyntactic (grammatical gender) and extralinguistic (speaker’s apparent gender) information. That said, we observed an atypical, wide distribution of ELAN effect over fronto-central and posterior regions, whereas a typical ELAN has a more left-frontal locus. We suggest that this non-canonical topography might be caused by a shift to the right hemisphere locations supporting speaker’s voice processing^53–55^. Indeed, whereas (morpho)syntactic processing is most often associated with left inferior-frontal areas^50^, the speaker’s voice identification is known to involve the right temporal lobe and the right inferior frontal cortex^56^, sources likely activated by the auditory stimulation with male and female voices presented in the current study. This simultaneous activation or a large bilateral network^57^ in the course of phrasal processing may have pushed the center of gravity of the overall scalp-surface potential distribution to more central areas. Nevertheless, taking into account the limited spatial resolution of the EEG technique, the localization of present ERP effects should be treated with caution. Future studies using other methodologies with a better spatial localization, such as MEG with individual MRI-based source reconstruction, are required to further explore the topography of the present effects putatively indexing the integration of extralinguistic information during speech processing.

To the best of our knowledge, this is the first demonstration of such an early ERP effect for extralinguistic information during morphosyntactic processing, with previous findings revealing only later effects during subsequent stages of sentence processing including syntax morphology and semantics^15–17,23^. At the same time, this finding is in line with previous studies reporting ELAN/sMMN effects for grammatical agreement violations and suggesting automatic (morpho)syntactic parsing even in the absence of attention to the linguistic input^30,40,41,44,58,59^. The crucial difference, however, is that the early agreement effect in the present study was elicited by voice-verb gender incongruencies in otherwise grammatically sound phrases, rather than by (morpho)syntactically infelicitous combinations used in previous studies. This suggests that the reported ELAN-like reflects the failure to integrate the two sources of information in cases of violation of the congruency between the speaker’s voice and the grammatical gender, as opposed to pure morphosyntactic violation processing known from previous studies.

A mechanistic explanation of early syntactic responses has been offered by the so-called (morpho)syntactic priming/prediction hypothesis^41^, claiming that preceding information (e.g., the subject in subject-verb phrases) leads to pre-activation of the relevant affix representation in order to facilitate and expedite input processing. Thus, when the affix finally arrives, it has been in part preactivated and less activation is necessary relative to pre-affix baseline. When such a pre-activation is not possible, the representation of the unprimed affix (or any other unexpected morpheme) has to be activated from a lower functional state, thus manifesting as a larger activation, relatively to the pre-affix baseline. Thus, a relatively larger response is registered for non-preactivated items in incongruent combinations. Whereas this original proposal was based on the existence of the associative links between morpheme representations (formed through their co-activation during previous language experience), it can now be extended to include extralinguistic information. In this view, concurrent processing of the first-person singular pronoun and speakers’ voice could pre-activate the related verb affix in pronoun–verb phrases. The response to the verbal gender affix is therefore connected with the preceding I-pronoun which itself triggers the effect of gender congruency from speaker’s voice. In this sense, we could hypothesize that the speaker’s voice (e.g., male) pre-activates expectations for the corresponding gender morphology (e.g., masculine). As a result, the speaker’s gender is used to automatically predict the upcoming verb’s morphology in order to maintain interlocutional alignment^46^. A very early chronometry of this response supports the idea of activating syntactic prediction templates (syntactic representation of a particular grammatical structure) which are available to the processor even before the input unequivocally supports this prediction^47^.

Importantly, one has to be cautious about the generalizability of the present findings obtained with just one type of voice/gender manipulation. Furthermore, whereas the present result specifically refers to the first-person phrases, other types of context-dependent pre-activation can be hypothesized for other constructions, which remains to be tested in future studies. Other aspects of the speaker-dependent information may be also pre-processed as particular language templates, and their violation should be accompanied by similarly early electrophysiological responses as suggested by some previous MMN studies (e.g.,^14^). Note, however, that such an early onset of the observed modulation could also reflect a high predictability of the stimuli with the voice gender identity being clear from the pronoun onset and before the gender-specific affix onset. While this interpretation does not undermine the reported results per se, further studies are necessary to further elucidate this. Such studies could use a larger choice of forms of different genders and possibly other morphosyntactic features, as well as different voices in both congruent and incongruent conditions with more stimulus variability. For example, there could be cases (e.g., quotations) whereby a male voice can report a sentence spoken in the first person with the feminine gender marker. We may expect a different response pattern in such cases with additional available context further modulating or even cancelling the present early effects, a possibility that should also be addressed in future experiments.

Another important aspect of our findings is the fact that the present ERP effect was found for verbal stimuli presented outside of attentional focus, as the participants were engaged in their primary visual task and did not pay attention to the linguistic input (as also validated by behavioral assessments showing that the subject complied with the primary task of watching a video and largely ignored the auditory input). This result reinforces the findings of other studies that documented grammatical morphosyntactic effects for unattended agreement violations^43,44,49^. One important difference, however, is that the effect reported here was elicited by extralinguistic rather than purely morphosyntactic factors. The early time-course of the effect and its emergence outside of the attentional focus indicate a high degree of automaticity of extralinguistic feature’s integration into sentence processing.

We have also registered a congruency effect at a later time window (350-450 ms), in the form of a more negative response for voice incongruent stimuli than for congruent ones. This effect, compatible with the N400 modulation, is similar to that previously observed for subject-gender agreement^23^. In line with previous studies, this N400-like effect may reflect the integration of extralinguistic information at a secondary stage of sentence processing^17,23^. Although auditory N400-like effects are typically distributed either bilaterally or with greater left hemispheric activation^60,61^, the modulation reported here showed right hemispheric topography; this is, nonetheless, in line with other studies reporting distributed right hemispheric activation^62,63^ (see for the N400 topography discussion^64^). In terms of its more anterior fronto-central distribution, this shift might result from verbal processing, which is known to lead to more frontal N400 effects^65^. Indeed, using other techniques with better spatial localization (fMRI), specific morphosyntactic processing has been observed bilaterally, and showed to relate to the activation of the inferior-frontal gyri^66^; however, the ERP methodology used here does not possess the spatial resolution necessary to confirm a similar localization of the present effect, which remains to be tested in future studies. Together with the overlap with preceding fronto-central negativity during pragmatic processing^67^, the atypical right fronto-central localization of N400 responses has been suggested to be connected with the different representation of stereotypical knowledge in comparison to general semantic knowledge^68^. Moreover, relatively more frontal N400-like effects have been previously reported for auditory stimulus presentation, such as implemented in the current study, also leading to longer latencies of the component in auditory domain^69–71^. Importantly, previous studies reporting N400 effects for extralinguistic integration found these responses during active linguistic processing (auditory attended presentation task) while the current study implemented passive presentation, also supporting the largely automatic nature of these effects.

Regarding the functional significance of the N400 effect, it has been specifically related to further integration of voice-gender information during the semantic stage of analysis. Indeed, the detection of a verbal affix violation may trigger the reanalysis of the preceding pronoun, but since in the current study a first-person pronoun was presented, which (unlike the third-person “*he/she*”) does not have gender feature, no syntactic re-evaluation could take place. Instead, listeners would likely start to re-evaluate the speaker’s voice gender which results in the N400-like effect^15,17^.

Overall, our findings of both early and late ERP effects, maximal around 150 and 400 ms after the onset of the verbal suffix, respectively, might suggest that the integration of speaker-dependent features into sentence processing likely unfolds over the initial few hundred milliseconds of speech processing, commencing at an early automatic interactive stage followed by a later re-integration stage. Importantly, although later N400 effects are often considered to reflect more controlled processing (see, e.g.,^29,44^, for automatic and controlled syntactic ERPs), the present study shows these effects can be also generated in a passive task using an attention-free design. To fully attest this putative automaticity of these processes, future studies should implement an explicit attention manipulation and directly compare brain’s responses to attended and unattended voice-syntax (in)congruences and including more gender agreement contrasts.

## Conclusion

The present ERP study provides electrophysiological evidence showing how extralinguistic information is integrated into the neural processing of syntactic information by the human brain. ERP analysis of brain responses to voice-congruent and voice-incongruent pronoun–verb phrases revealed two processing stages: an early automatic stage (reflected in an ELAN-like time window) and a later stage (in an N400-like time window), both showing enhanced fronto-central negativity for the mismatching stimuli. These findings (1) confirm previous results and, (2) provide novel evidence regarding the neurophysiological bases of syntactic processing. Importantly, we show that the latter does not only rely on the available linguistic information but also on the presence of extralinguistic cues, such as the speaker’s gender information assumed from their voice. Moreover, this integration appears to take place in a rapid and automatic fashion as evident from the timing of the effects and their presence in the absence of focused attention on the stimuli. Nevertheless, further investigations are necessary in order to better understand the neural bases of this interaction. The use of more complex paradigms, including other morphosyntactic contrasts and voice variability, as well as the use of brain imaging techniques with high resolution at both temporal and spatial dimensions (such as MEG with MR-based source reconstruction) will lead to a better understanding of spoken language comprehension in the human brain.

## Supplementary Information

Supplementary Information 1.

Supplementary Information 2.

Supplementary Information 3.

Supplementary Information 4.

Supplementary Information 5.

Supplementary Information 6.

## Data Availability

The datasets generated during and/or analyzed during the current study are available from the corresponding author on reasonable request.
