# Supplementary material for: Neurophysiological correlates of automatic integration of voice and gender information during grammatical processing
Source: Sci Rep. 2022 Jul 30;12:13114. doi: 10.1038/s41598-022-14478-2 (PMC9339001; doi:10.1038/s41598-022-14478-2)
Supplement: Supplementary file 1 — Supplementary Information 1. [file 41598_2022_14478_MOESM1_ESM.docx]

Appendix A. Full set of verbs used in phrasal stimuli.

| **Verb in masculine form** | **Form frequency, log** | **Bigram frequency, log** |
| --- | --- | --- |
| popal ([pɐˈpal], gotmasc) | 4.21 | 4.0 |
| velel ([vʲɪˈlʲel], orderedmasc) | 4.13 | 4.0 |
| kupil ([kʊpʲˈil]*,* boughtmasc) | 3.99 | 3.87 |
| polil([pɐˈlʲil], wateredmasc) | 3.98 | 3.96 |
| pozhal ([pɐˈʐal]*,* shookmasc) | 3.93 | 3.96 |
| symel ([sʊˈmʲel], couldmasc) | 3.87 | 3.78 |
| nadel ([nɐˈdʲel], put onmasc) | 3.75 | 3.63 |
| pobil ([pɐˈbʲil], brokemasc) | 2.91 | 2.78 |
| zapel ([zɐˈpʲel], sangmasc) | 2.43 | 2.53 |
| zasel ([zɐˈsʲel], satmasc) | 2.3 | 2.2 |
