# Supplementary material for: Neurophysiological correlates of automatic integration of voice and gender information during grammatical processing
Source: Sci Rep. 2022 Jul 30;12:13114. doi: 10.1038/s41598-022-14478-2 (PMC9339001; doi:10.1038/s41598-022-14478-2)
Supplement: Supplementary file 2 — Supplementary Information 2. [file 41598_2022_14478_MOESM2_ESM.docx]

Appendix B. Full set of stimulus phrases.

| **Stimuli** | **Duration Male voice (s)** | **Duration Female voice (s)** |
| --- | --- | --- |
| ja ([ja], I) | 0.348 | 0.348 |
| pause between pronoun and verb | 0.076 | 0.076 |
| -l([l]; past tense suffix) | 0.086 | 0.086 |
| ja popal ([ja pɐˈpal], I gotmasc) | 0.903 | 0.964 |
| ja velel ([ja vʲɪˈlʲel], I orderedmasc) | 0.892 | 0.928 |
| ja kupil ([ja kʊpʲˈil], I boughtmasc) | 0.782 | 0.937 |
| ja polil ([ja pɐˈlʲil], I wateredmasc) | 0.748 | 0.946 |
| ja pozhal ([ja pɐˈʐal], I shookmasc) | 0.851 | 0.929 |
| ja sumel ([ja sʊˈmʲel], I couldmasc) | 0.927 | 1.061 |
| ja nadel ([ja nɐˈdʲel], I put onmasc) | 0.942 | 1.038 |
| ja pobil ([ja pɐˈbʲil], I brokemasc) | 0.812 | 0.960 |
| ja zapel ([ja zɐˈpʲel], I sangmasc) | 0.943 | 1.061 |
| ja zasel ([ja zɐˈsʲel], I satmasc) | 0.978 | 1.032 |
