# Supplementary material for: Neurophysiological correlates of automatic integration of voice and gender information during grammatical processing
Source: Sci Rep. 2022 Jul 30;12:13114. doi: 10.1038/s41598-022-14478-2 (PMC9339001; doi:10.1038/s41598-022-14478-2)
Supplement: Supplementary file 3 — Supplementary Information 3. [file 41598_2022_14478_MOESM3_ESM.docx]

Appendix C. Post-hoc tests for ELAN-like time window for 64 channels used in the original analysis: congruent vs. incongruent conditions. Only channels with significant post-hoc test results are listed.

| Channel | Mean difference | Std.Error | Sig. |  |
| --- | --- | --- | --- | --- |
| FFC1h | .237 | .105 | .03 | * |
| FFC2h | .2 | .096 | .044 | * |
| FC1 | .266 | .117 | .029 | * |
| FC2 | .291 | .124 | .024 | * |
| FCC1h | .188 | .09 | .045 | * |
| FCC2h | .186 | .08 | .026 | * |
| **C1** | **.227** | **.073** | **.004** | ******* |
| C2 | .199 | .074 | .011 | ** |
| C4 | .175 | .085 | .046 | * |
| CCP3h | .21 | .082 | .014 | ** |
| CCP1h | .189 | .075 | .017 | ** |
| CCP2h | .173 | .066 | .013 | ** |
| CCP4h | .178 | .079 | .031 | * |
| CP1 | .177 | .079 | .032 | * |
| CP2 | .171 | .075 | .027 | * |
