# Supplementary figures and images for: Neurophysiological correlates of automatic integration of voice and gender information during grammatical processing
Source: Sci Rep. 2022 Jul 30;12:13114. doi: 10.1038/s41598-022-14478-2 (PMC9339001; doi:10.1038/s41598-022-14478-2)

Appendix D. ERP waveforms of the topographical clusters in 130-170 ms time window.


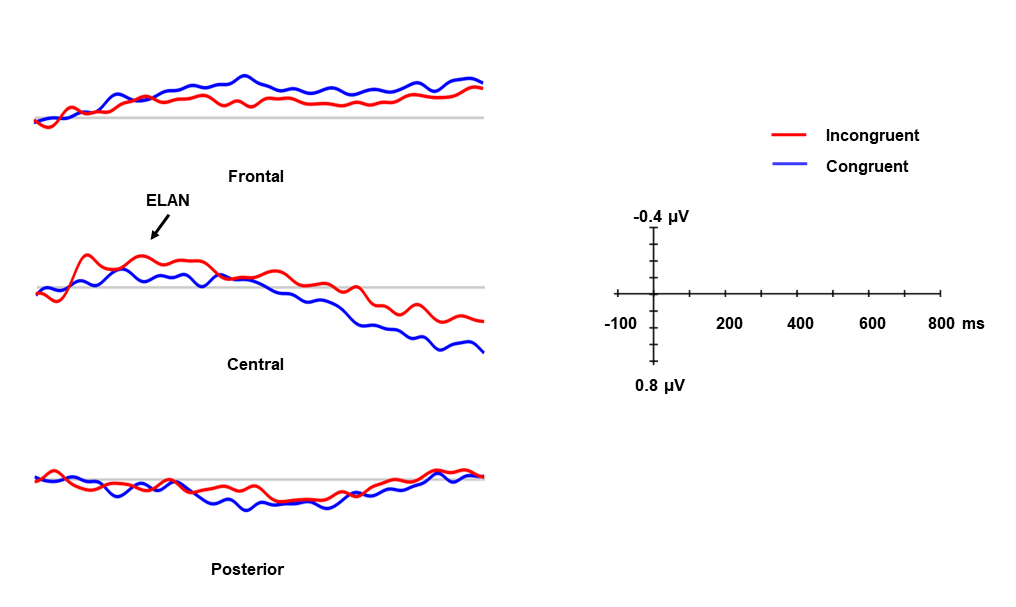

Supplement: Supplementary file 4 — Supplementary Information 4. [file 41598_2022_14478_MOESM4_ESM.docx]

Appendix F. ERP waveforms of the topographical clusters in 350-450 ms time window.


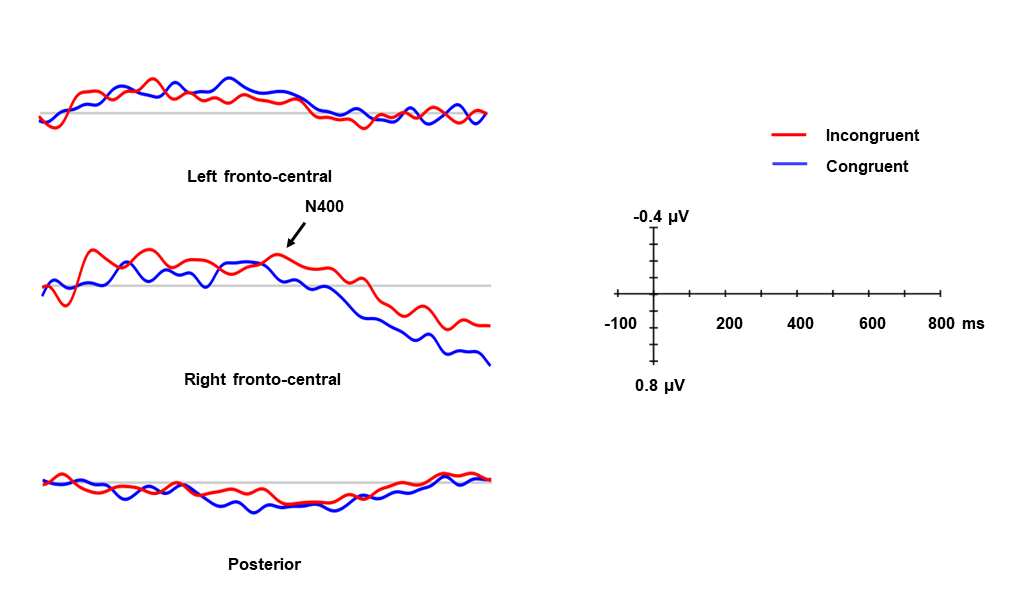

Supplement: Supplementary file 6 — Supplementary Information 6. [file 41598_2022_14478_MOESM6_ESM.docx]
