# Supplementary material for: Neurophysiological correlates of automatic integration of voice and gender information during grammatical processing
Source: Sci Rep. 2022 Jul 30;12:13114. doi: 10.1038/s41598-022-14478-2 (PMC9339001; doi:10.1038/s41598-022-14478-2)
Supplement: Supplementary file 5 — Supplementary Information 5. [file 41598_2022_14478_MOESM5_ESM.docx]

Appendix E. Post-hoc tests for ELAN-like time window for 64 channels used in the original analysis: congruent vs. incongruent conditions. Only channels with significant post-hoc test results are listed.

| Channel | Mean difference | Std.Error | Sig. |  |
| --- | --- | --- | --- | --- |
| FC4 | .194 | .084 | .027 | ** |
| FCC4h | .223 | .097 | .027 | ** |
| FCC6h | .158 | .072 | .034 | * |
| C4 | .205 | .098 | .043 | * |
